# Tonsil-derived Mesenchymal Stem Cells Ameliorate CCl_4_–induced Liver Fibrosis in Mice via Autophagy Activation

**DOI:** 10.1038/srep08616

**Published:** 2015-02-27

**Authors:** Minhwa Park, Yu-Hee Kim, So-Youn Woo, Hye Jin Lee, Yeonsil Yu, Han Su Kim, Yoon Shin Park, Inho Jo, Joo-Won Park, Sung-Chul Jung, Hyukjin Lee, Byeongmoon Jeong, Kyung-Ha Ryu

**Affiliations:** 1 Department of Microbiology, Ewha Womans University School of Medicine, 1071 Anyangcheon-ro Yangcheon-gu, Seoul, 158-710, Korea; 2 Department of Otorhinolaryngology–Head and Neck Surgery, Ewha Womans University School of Medicine, 1071 Anyangcheon-ro Yangcheon-gu, Seoul, 158-710, Korea; 3 Department of Molecular Medicine, Ewha Womans University School of Medicine, 1071 Anyangcheon-ro Yangcheon-gu, Seoul, 158-710, Korea; 4 Department of Biochemistry, Ewha Womans University School of Medicine, 1071 Anyangcheon-ro Yangcheon-gu, Seoul, 158-710, Korea; 5 Department of Pharmaceutics, Ewha Womans University, 52 Ewhayeodae-gil, Seodaemun-gu, Seoul, 120-750, Korea; 6 Department of Chemistry and Nano Science, Ewha Womans University, 52 Ewhayeodae-gil, Seodaemun-gu, Seoul, 120-750, Korea; 7 Department of Pediatrics, Ewha Womans University School of Medicine, 1071 Anyangcheon-ro Yangcheon-gu, Seoul, 158-710, Korea; 8 Ewha Global Top 5 Research Program

## Abstract

Liver transplantation is the treatment of choice for chronic liver failure, although it is complicated by donor shortage, surgery-related complications, and immunological rejection. Cell transplantation is an alternative, minimally invasive treatment option with potentially fewer complications. We used human palatine tonsil as a novel source of mesenchymal stem cells (T-MSCs) and examined their ability to differentiate into hepatocyte-like cells *in vivo* and *in vitro*. Carbon tetrachloride (CCl_4_) mouse model was used to investigate the ability of T-MSCs to home to the site of liver injury. T-MSCs were only detected in the damaged liver, suggesting that they are disease-responsive. Differentiation of T-MSCs into hepatocyte-like cells was confirmed *in vitro* as determined by expression of hepatocyte markers. Next, we showed resolution of liver fibrosis by T-MSCs via reduction of TGF-β expression and collagen deposition in the liver. We hypothesized that autophagy activation was a possible mechanism for T-MSC-mediated liver recovery. In this report, we demonstrate for the first time that T-MSCs can differentiate into hepatocyte-like cells and ameliorate liver fibrosis via autophagy activation and down-regulation of TGF-β. These findings suggest that T-MSCs could be used as a novel source for stem cell therapy targeting liver diseases.

Mesenchymal stem cells (MSCs) can be differentiated into a variety of tissue-specific lineages and have been demonstrated to possess immune regulatory function^1^,^2^,^3^. Bone marrow (BM) and adipose tissue are the major sources of MSCs that have been identified so far; however, their use in clinical application is limited due to the low cell yields and the invasive procedures required to isolate these cells^4^. Therefore, it would be desirable to find alternative and non-invasive sources of MSCs. Tonsillectomy is the most common operative procedure for tonsil hyperplasia and inflammation, and the removed tissue is usually discarded^5^,^6^. Along with others, we have isolated MSCs from palatine tonsils (T-MSCs) and shown that they undergo mesodermal differentiation and possess immunomodulatory effects^7^,^8^,^9^. T-MSCs have been proposed as an alternative source of adult stem cells given that they are readily available as surgically removed “waste tissue”.

Allogeneic liver transplantation is the only effective treatment available for liver failure; however, donor organs are in short supply^10^,^11^. Although liver-derived hepatocyte transplantation is a potential alternative to organ transplantation, this method still relies upon the availability of appropriate donor tissue. Therefore, non-organ sources of hepatocytes are being explored for use in cell therapies, with one possible source being adult stem cells. It has been reported that BM-MSC transplantation is effective for ameliorating liver injury in a mouse model, which appears to be mediated by the differentiation of BM-MSCs into hepatocyte-like cells^12^,^13^. However, the underlying mechanisms of action of MSCs are not well understood.

A major characteristic of hepatic fibrosis is excessive accumulation of extracellular matrix (ECM) proteins and activation of hepatic stellate cells (HSCs). Dysregulation in ECM turnover due to the activation of HSCs has been observed in fibrotic progression^14^,^15^. Activated HSCs produce collagens as well as tissue inhibitors of metalloproteinases (TIMPs 1 and 2) that prevent collagen degradation^16^,^17^, thus promoting the progression of liver fibrosis. Among many factors that drive fibrosis, transforming growth factor-β (TGF-β) has been recognized as a major mediator that increases collagen expression and activates HSCs^18^,^19^,^20^,^21^. Recently, autophagy, a process by which cells degrade and recycle proteins, has been suggested to be involved in fibrosis by modulating TGF-β expression^22^,^23^.

In this study, we examined the effects of T-MSCs in an acute liver injury. We used carbon tetrachloride (CCl_4_)-induced liver injury in a mouse model because CCl_4_ specifically targets the liver by producing a free radical (CCl_3_), leading to lipid peroxidation, hydropic degeneration, steatosis, and hepatocellular zone 3 necrosis^24^,^25^. Herein, we showed that T-MSCs have a regenerative effect by migrating to the site of liver injury and differentiating into hepatocyte-like cells. We also demonstrated that T-MSCs exert this effect by down-regulating TGF-β expression through autophagy activation. Therefore, our findings provide the first evidence of the therapeutic potential of human T-MSCs for the treatment of liver fibrosis.

## Results

### Phenotype and differentiation potential of T-MSCs

**Figure 1:**
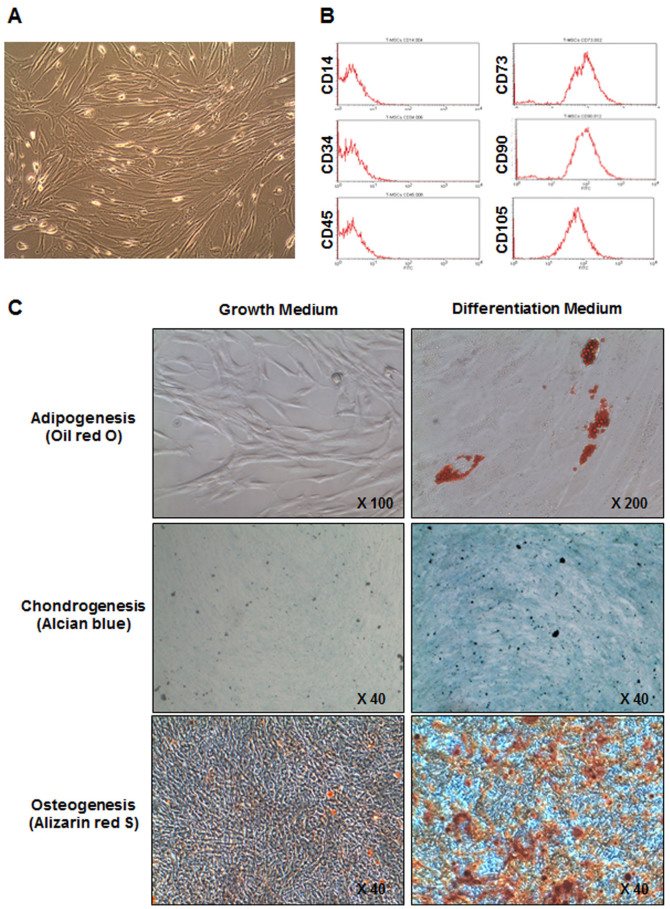
Phenotype and differentiation potential of tonsil-derived mesenchymal stem cells (T-MSCs) in culture. Morphological appearance of T-MSCs at day 3 (A). Flow cytometry analysis of cell surface markers of T-MSCs (B). Representative images of adipocyte, chondrocyte and osteocyte differentiation of T-MSCs cultured in the growth or differentiation medium (C).

T-MSCs proliferated as spindle-shaped adherent cells when examined after 3 days of culture (day 3) (Figure 1A). Staining for surface markers revealed that T-MSCs were negative for hematopoietic cell markers (CD14, CD34, and CD45) and positive for primitive cell markers (CD73, CD90, and CD105) at day 3 (Figure 1B). We also investigated the potential of T-MSCs to differentiate into cells of mesodermal origin by culturing them in differentiation culture medium for 3 weeks. Adipogenic differentiation was evaluated by lipid droplet formation, chondrogenic differentiation by sulfated proteoglycan staining, and osteogenic differentiation by matrix mineralization. Results showed a multipotency of T-MSCs (Figure 1C).

### T-MSCs selectively migrated into the CCl_4_-injured liver

**Figure 2:**
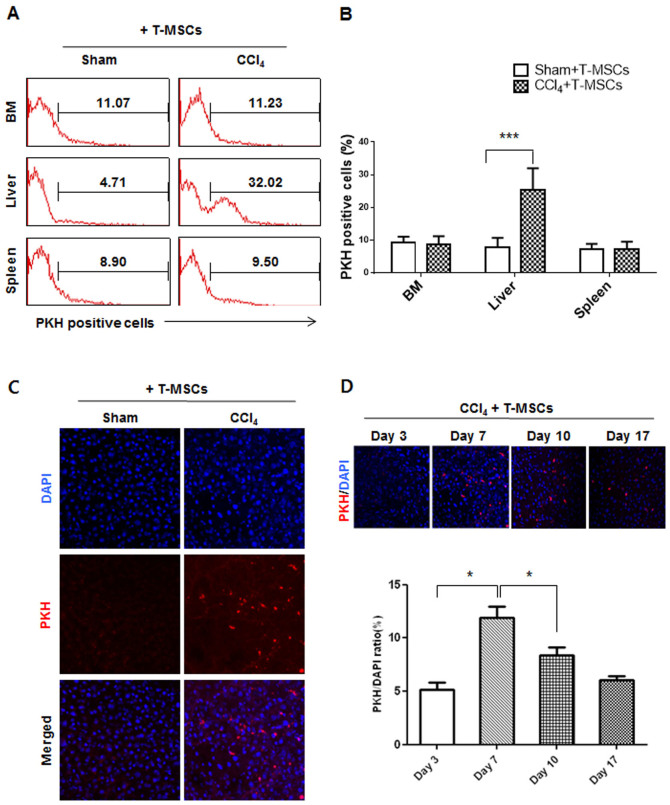
*In vivo* localization of T-MSCs in CCl_4_-injured liver. Representative flow cytometry histograms of PKH-positive cells from five different mice; T-MSCs in bone marrow (BM), liver, and spleen at 7 days post-infusion (A). Statistical analysis of the percentage of PKH-positive cells in BM, liver, and spleen by flow cytometry (B). Localization of T-MSCs in the CCl_4_-injected mouse liver using IF at day 7 post-infusion (C). Changes in the number of T-MSCs present at days 3, 7, 10, and 17 post-infusion, and the quantification of PKH-positive area per field of 100 DAPI-labeled cells (average of 7–12 fields) (D). The data are expressed as the means ± SEM and data were analyzed by one-way or two-way ANOVA (*P < 0.5, ***P < 0.001).

Following an infusion into untreated (sham) and CCl_4_-treated mice, we monitored the distribution of transplanted T-MSCs using a PKH26, a fluorescent cell marker^26^. We observed selective recruitment of T-MSCs into the CCl_4_-injured liver (4.7 ± 3.0% in sham versus 32.0 ± 3.3% in CCl_4_ mice) using flow cytometry. Infused cells were not detected in BM (11.0 ± 1.8 in sham versus 11.2 ± 2.6 in CCl_4_ mice) or spleen (8.9 ± 1.8 in sham versus 9.5 ± 4.5 in CCl_4_ mice) (Figure 2A). The increase in the number of migrated T-MSCs in the liver of CCl_4_-treated mice was statistically significant compared to the liver of the control mice (*P* < 0.001) (Figure 2B). Recruitment of T-MSCs into the liver was also examined by immunofluorescence (IF) and again showed that T-MSCs only migrated into the site of injury (Figure 2C). Next, we examined the changes in the number of T-MSCs present in the damaged liver on days 3, 7, 10, and 17. Results showed that the homing of T-MSCs to the site of injury was at its greatest 1 week after infusion, and then gradually declined (Figure 2D). Thereafter, we decided to analyze the data at 7 days post-infusion.

### T-MSCs recruited into the injured liver differentiated into hepatocyte-like cells

**Figure 3:**
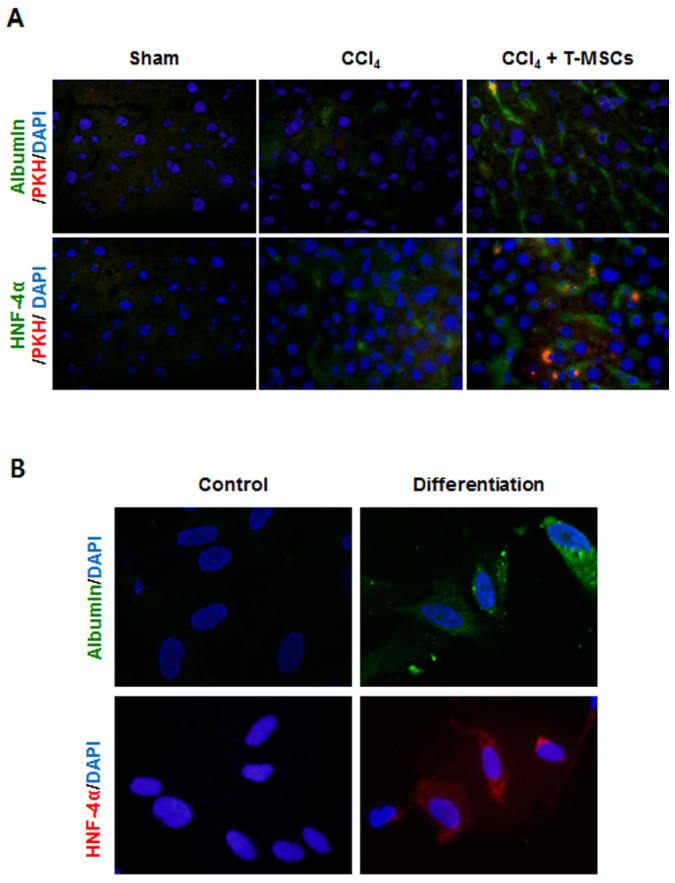
Expression of hepatocyte markers in differentiated T-MSCs. Determination of albumin and HNF-4α expression in the mouse liver using IF (A). Detection of albumin and HNF-4α expression in differentiated T-MSCs by IF (B).

In order to identify whether T-MSCs recruited into the injured liver differentiated into hepatocyte-like cells, we stained mouse liver samples with albumin and HNF-4α. As shown in Figure 3A, there was an increase in the numbers of albumin and HNF-4α positive cells at 7 days post-infusion, which suggested that T-MSCs recruited into the CCl_4_-injured liver differentiated into hepatocyte-like cells. To confirm this possibility *in vitro*, we cultured T-MSCs in differentiation medium containing insulin-like growth factor (IGF), hepatocyte growth factor (HGF), dexamethasone, and oncostatin M (OSM) to induce the commitment of T-MSCs to the hepatic lineage. After 3 weeks of culture in differentiation medium, expression of albumin and HNF-4α was examined using IF staining. Results confirmed the differentiation of T-MSCs into hepatocyte-like cells (Figure 3B).

### CCl_4_-induced liver fibrosis was ameliorated by T-MSCs injection

**Figure 4:**
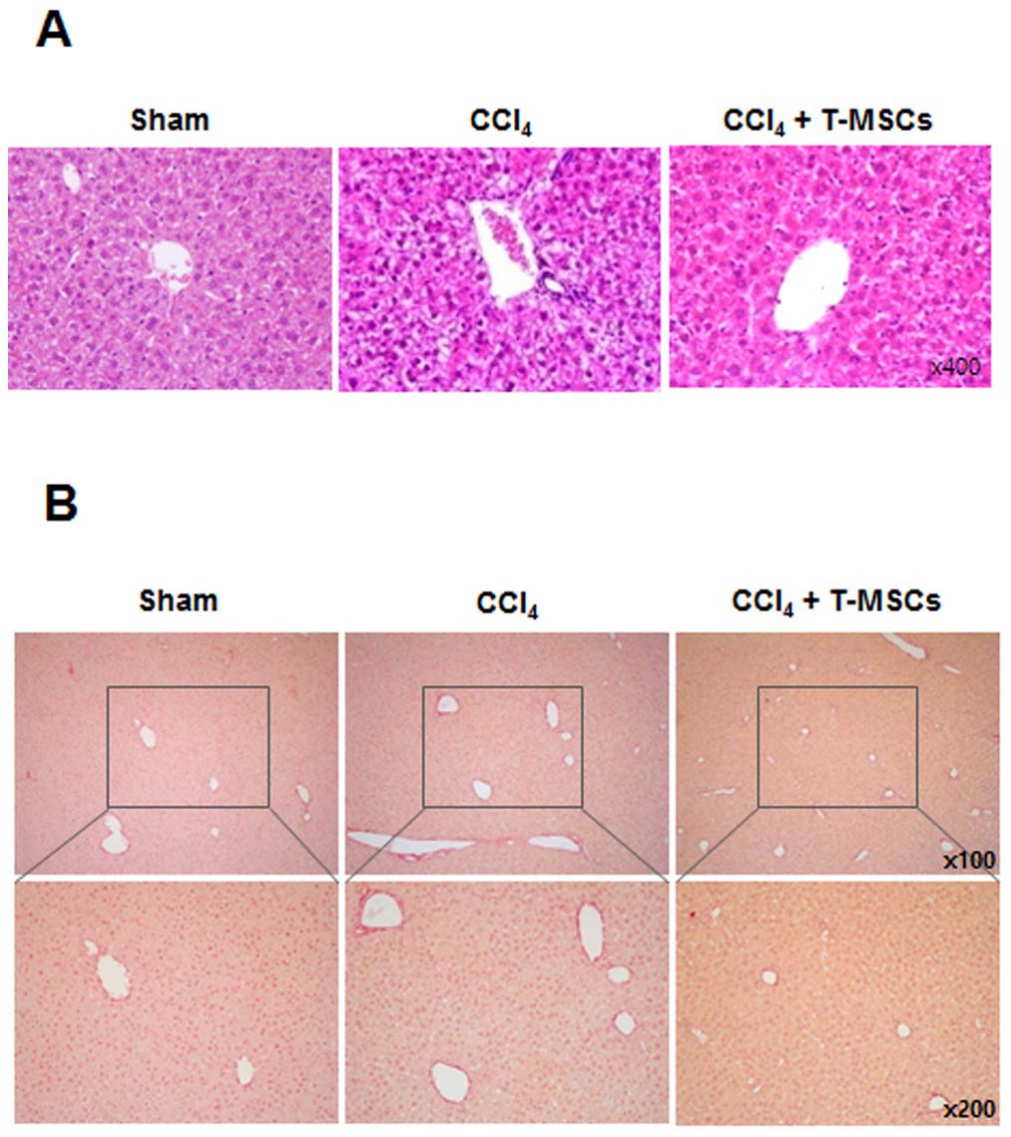
T-MSC infusion ameliorated liver fibrosis in CCl_4_-injured mice. H&E staining for liver histology (A) and Sirius Red staining to detect collagen (B).

To assess whether recruited T-MSCs could ameliorate liver damage, we analyzed liver histology of CCl_4_-injured mice after infusion with or without T-MSCs. Using hematoxylin and eosin (H&E) staining, we observed that necrosis due to CCl_4_ treatment was reversed by T-MSCs infusion (Figure 4A). We also measured liver fibrosis using Sirius Red staining and found reduced collagen deposition around the central vein in the livers of CCl_4_–treated mice after T-MSCs infusion (Figure 4B).

### The anti-fibrotic effect of T-MSCs is due to autophagy activation and reduction of TGF-β expression

**Figure 5:**
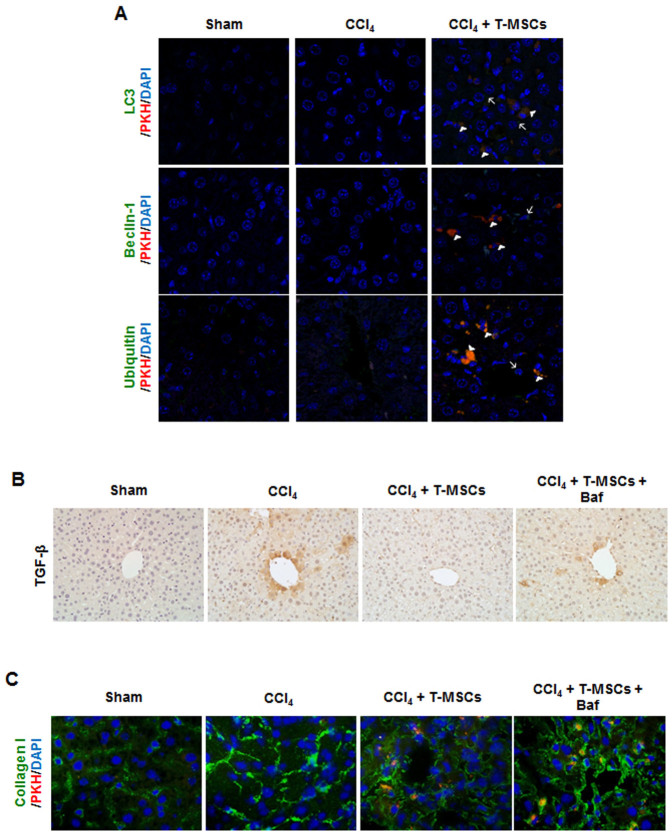
Autophagy induced by T-MSCs reduced TGF-β and type I collagen expression in the CCl_4_-damaged liver. Confocal images of LC3, beclin-1, and ubiquitin expression in liver of control, CCl_4_-treated, and CCl_4_/T-MSC-treated mice. Autophagy detected in parenchymal cells is indicated using arrows and in T-MSCs using arrow heads (A). Bafilomycin A1 (Baf) was injected with T-MSCs to inhibit autophagy. Expression of TGF-β (B) and type I collagen (C) were examined in mouse liver using IHC and IF, respectively.

Autophagy was examined in order to identify the mechanism of action of T-MSCs on liver regeneration. We stained liver tissue for the detection of LC3, beclin-1, and ubiquitin, showing that these autophagy-related proteins were expressed in T-MSCs and parenchymal cells from the CCl_4_-injured mice (Figure 5A). Next, we examined the expression of TGF-β, which plays a pivotal role in the progression of fibrosis. We found that the increased TGF-β expression in the CCl_4_-treated group was not observed in the CCl_4_/T-MSCs-treated group. Furthermore, the effect of T-MSCs on TGF-β down-regulation was blocked by treatment with the autophagy inhibitor, bafilomycin A1 (Figure 5B).

Given that TGF-β signaling induces collagen synthesis, we examined type I collagen deposition in liver tissue using IF. Data showed that increased collagen I in the liver of the CCl_4_-injured group was reduced by T-MSCs transplantation. Autophagy inhibition again neutralized the effect of T-MSCs (Figure 5C).

## Discussion

In this report, we showed the effects of T-MSCs on liver regeneration. First, we demonstrated the selective recruitment of T-MSCs into the injured liver, and their differentiation into albumin-expressing, hepatocyte-like cells. Second, we observed the reduction of liver fibrosis following T-MSCs infusion. Our findings indicated that the mechanism of action of T-MSCs in resolution of acute liver damage is via an accumulation of autophagy-related proteins and inhibition of TGF-β.

Previously, we have demonstrated the recruitment of T-MSCs into damaged organs using a graft-versus-host disease (GVHD) mouse model^27^. In that study, we showed that the MIP-2/CXCR2 axis plays a role in the pathogenesis of GVHD by recruiting T lymphocytes into the GVHD target organs^28^. Given that T-MSCs secrete large amounts of CXCR2 ligand proteins such as IL-8, CXCL1, and CXCL5 (manuscript in preparation), CXCR2 may participate in the homing of T-MSCs into the injured liver. It is also suggested that HGF/c-Met signaling may be involved in the recruitment of MSCs into the damaged liver^29^.

Interestingly, the localization of T-MSCs in the damaged liver reached its peak at 7 days post-injection, and then gradually decreased. It is possible that T-MSCs migrated to other organs or died after day 7. In addition, T-MSCs infused into normal mice were not detected in the organs that were examined, suggesting that they may have been cleared or migrated to other organs that we did not evaluate. It was reported that BM-MSCs migrated into the salivary gland when infused into the normal and salivary gland-damaged mice^30^. While we did not observe the localization of T-MSCs into the liver of non-injured mice, this could be due to differences in chemokine receptor profiles between stem cell populations. Therefore, it remains to be elucidated whether the infused T-MSCs migrated to other sites in the normal or CCl_4_-injured mice after day 7, or were simply cleared. Additionally, it will be important to determine the chemokine receptor profiles of T-MSCs, as well as their longevity *in vivo*.

Regarding the fate of the injected cells, we previously reported that BM-derived MSCs could differentiate into hepatocyte-like cells *in vitro* as well as *in vivo*^31^. Furthermore, others have demonstrated the expression of human type albumin in the host liver as an indicator of functional restoration of damaged liver after xenotransplantation^32^. Therefore, we investigated whether injected T-MSCs could differentiate into albumin-expressing hepatocyte-like cells. Following incubation in differentiation medium containing IGF, HGF, OSM, and dexamethasone^31^,^33^, T-MSCs were shown to differentiate into hepatocyte-like cells. Expression of the hepatic parenchymal markers albumin and HNF-4α^31^,^34^ showed that our T-MSC cultures contained functional hepatocytes.

Hepatic fibrosis is a progressive pathological process involving multicellular and molecular events that ultimately lead to the deposition of excess ECM proteins such as collagens^35^. This study evaluated fibrosis using Sirius Red staining and IF for type I collagen. We found that collagen accumulation was significantly decreased following T-MSCs transplantation. We also demonstrated that the expression of TGF-β was down-regulated by T-MSC injection. These findings provide evidence that T-MSCs ameliorated liver fibrosis in this mouse model. It is generally accepted that HSCs are central to the process of fibrosis and are a major source of ECM components^36^,^37^. Upon activation, HSCs attain a myofibroblastic phenotype and produce ECM proteins, including enzymes such as matrix metalloproteinases (MMPs) and tissue inhibitors of metalloproteinases (TIMPs). Therefore, it will be important to determine whether MSCs interact with HSCs directly or indirectly, and whether their interaction is involved in the resolution of liver fibrosis.

The potential use of stem cell therapy for the treatment of liver fibrosis is promising and its role in tissue regeneration has been extensively studied^9^,^29^,^38^. It has been suggested that autophagy may play a role in the regenerative potential of stem cell therapy^9^,^39^. Jung et al. reported that the mechanism by which human placenta-derived MSCs promote tissue regeneration may involve HIF-alpha and autophagy, with autophagy being induced in CCl_4_-damaged hepatic cells in a hypoxia-dependent manner^9^. Autophagy activation appears to be involved in T-MSC-mediated tissue regeneration as well, as shown by an increase in LC3, beclin-1, and ubiquitin expression. Inhibition of autophagy blocked the effects of T-MSCs on down-regulation of TGF-β and type I collagen expression. These results corroborate other studies that have shown the involvement of autophagy in the regulation of TGF-β expression and type I collagen synthesis^22^,^23^.

Our results indicate that T-MSCs can be a novel source of adult stem cells. T-MSCs possess the advantage over BM-MSCs or Ad-MSCs in that they can be easily obtained from “waste” tissue. However, more detailed analyses are required before T-MSCs can be developed as an alternative source for stem cell therapy, such as testing the efficacy of T-MSCs compared with other sources of adult stem cells for the treatment of disease.

In summary, we have shown that T-MSCs home into the CCl_4_-damaged liver and differentiate into hepatocyte-like cells. We have also demonstrated that T-MSCs can repair liver injury induced by CCl_4_ via autophagy activation and inhibition of TGF-β expression. This study would be the first to suggest that T-MSCs could be used in stem cell therapy as an alternative treatment for liver fibrosis.

## Methods

### Ethics statement and human samples

Human tonsils were obtained from tonsillectomies performed in the Department of Otorhinolaryngology, Head and Neck Surgery at Ewha Womans University Mok-Dong Hospital. The protocol was approved by the research ethics committees of Ewha Womans University Mok-Dong Hospital (IRB #ECT11-53-02). Written informed consent was obtained from all patients.

Experiments and procedures were approved by the Animal Ethics Committee at Ewha Womans University College of Medicine (ESM #11-0185) and all experiments were performed in accordance with the approved guidelines and regulations. Six- to eight-week-old female C57BL/6 mice (Oriental Bio, Seongnam, Korea) were housed at 21–23°C and 51%–54% humidity with a 12-hr light/dark cycle, and supplied with food and water *ad libitum*.

### Isolation of T-MSCs and cell culture

Tonsils were washed three times with phosphate-buffered saline (PBS) in 50 ml conical tubes, cut into ~5 mm pieces, and incubated with collagenase type I (210 U/mL; Gibco BRL, Carlsbad, CA, USA) and DNase I (10 μg/mL; Sigma-Aldrich, St. Louis, MO, USA) in 10 mL Dulbecco's Modified Eagle's Medium (DMEM, Welgene, Daegu, Korea) for 30 min at 37°C with stirring.

Cells were harvested by filtration through a cell strainer (pore size 70 μm; SPL, Pocheon, Korea) and washed twice by centrifugation (300 × g for 3 min at room temperature); cell pellets were resuspended with DMEM containing 20% heat-inactivated fetal bovine serum (FBS) for the first wash and with DMEM containing 10% FBS for the second. Mononuclear cells (MNCs) were isolated by centrifugation (300 × g for 30 min at room temperature) through a Ficoll-Paque^TM^ PREMIUM gradient (GE Healthcare, Pittsburgh, PA, USA). MNCs were plated at a density of 1 × 10^8^ cells per T-150 flask in DMEM supplemented with 10% FBS, 100 μg/mL streptomycin, and 100 U/mL penicillin (DMEM growth medium). Non-adherent cells were removed after 48 hrs by gentle pipetting and washing with PBS, and the remaining adherent cells were cultured in DMEM growth medium and sub-cultured twice per week.

### Mesodermal differentiation of T-MSCs

Adipogenic, chondrogenic, and osteogenic differentiation was induced by culturing T-MSCs for 3 weeks in appropriate culture medium purchased from Invitrogen. Verification of cell populations was carried out by staining 3-week culture samples. T-MSCs cultured with adipogenic medium were fixed in 4% PFA and stained with Oil Red O staining solution for 10 min at room temperature. After inducing chondrogenic differentiation, T-MSCs were fixed in 4% PFA and cartilage formation was examined by incubating cells with Alcian blue staining solution overnight at room temperature in the dark. T-MSCs that underwent osteogenic differentiation were fixed with 60% isopropyl alcohol then stained with Alizarin Red S solution (pH 4.2) for 3 min at room temperature. For visualization, cells were examined under a phase-contrast microscope.

### Cell labeling with PKH26

After 3–5 passages, T–MSCs were labeled with the PKH26 MINI kit (Sigma-Aldrich) according to the manufacture's protocol. Briefly, cell suspension (1 × 10^7^ cells/mL) was added to the dye solution (final concentration of 2 × 10^−6^ M of PKH26) and immediately mixed by pipetting. After incubating for 5 min at room temperature, an equal volume of FBS was added, and cells were allowed to incubate for 1 min. Cells were pelleted by centrifugation for 10 min (300 × g at room temperature), washed three times by resuspension in the same volume of DMEM growth medium, followed by final resuspension in 2 mL of PBS for mouse injection.

### Flow cytometry

T-MSCs were incubated with the following primary antibodies: FITC-conjugated anti-CD45 (2D1, mouse IgG1; BD Biosciences, Franklin Lakes, NJ, USA), PE-conjugated anti-CD14 (MφP9, mouse IgG_2b_; BD Biosciences), FITC-conjugated anti-human CD34 (BIRMAK3, mouse IgG_1_; IBGRL, Bristol, UK), PE-conjugated anti-human CD73 (AD2, mouse IgG_1_; BioLegend, San Diego, CA, USA), FITC-conjugated anti-human CD90 (5E10, mouse IgG_1_; BD Biosciences) and FITC-conjugated anti-human CD105 (266, mouse IgG_1_; BD Biosciences).

For PKH-positive cells, livers and spleens were collected from mice on day 7 after cell transfusion, cut into 1 cm^3^ pieces, rinsed with 5 mL PBS, chopped with scissors, and incubated in DMEM containing 100 μL/mL of collagenase D (Roche, Indianapolis, IN, USA) for 20 min at 37°C. Cell suspensions were collected and filtered through a cell strainer after the cells were centrifuged and resuspended twice with DMEM containing 10% FBS. Surface phenotype markers and PKH-positive cells were analyzed on a flow cytometer (FACSCalibur, BD Biosciences) and Cell Quest software (BD Biosciences).

### Liver injury model and cell transfusion

Acute liver injury was induced using CCl_4_ (Sigma-Aldrich). C57BL/6 mice were randomly divided into two groups: no treatment and CCl_4_-injured; each group contained 5 mice. Liver-injuries were induced by intraperitoneal injection of CCl_4_ (10% solution in olive oil, 10 μL/g of body weight) on 2 consecutive days. T-MSCs were infused (2 × 10^6^ per mouse) via tail vein injection on the following day and mice were sacrificed after 7 days post-infusion.

### Immunofluorescence staining

Liver tissues were cryopreserved in optimal cutting temperature medium (OTC, TissueTek, SakuraAmericas, Torrence, CA, USA). Frozen tissue sections (5 μm) were collected on glass slides and fixed with 100% methanol for 10 min at 4°C, then washed in PBS for 10 min at room temperature. For *in vitro* differentiated cells, cells were moved to Lab-TekTMII-CC2TM chamber slide (Nalgene Nunc International, Rochester, NY, USA) on day 22 and incubated overnight at 37°C, followed by fixation with 4% PFA for 10 min at 4°C. Slides were rinsed three times with PBS for 5 min each.

Paraffin-embedded sections were de-paraffinized in xylene for 10 min and dehydrated in 100%, 95%, 90%, 80% and 75% ethanol, 3 min in each. Slides were then washed with PBS for 7 min. Enzyme-induced antigen retrieval was performed using 1× PBS containing 0.1% Trypsin for 30 min at 37°C. Slides were fixed with ice-cold methanol for 10 min at room temperature and washed twice with ice-cold PBS. PBS containing 0.25% Triton X-100 was used as permeabilization solution for intracellular staining. After blocking with 1% bovine serum albumin (BSA) in 0.02% Tween-20 in PBS (PBST), slides were incubated in a humidified chamber for 1 hr at room temperature with the following primary antibodies in 1% BSA in PBST: anti-albumin (1:100, Abcam; Cambridge, MA, USA), anti-HNF-4α (1:50, Santa Cruz Biotechnology; Dallas, TX, USA), anti-LC3 (1:50, Proteintech; Chicago, IL, USA), anti-beclin-1 (1:50, Proteintech), anti-ubiquitin (1:25, Cell Signaling Technology; Danvers, MA, USA), anti-TGF-β (1:100, Abcam), and anti-collagen I (1:500, Abcam). Slides were then washed three times in PBS for 5 min and incubated with secondary antibodies in 1% BSA in PBST: FITC-conjugated anti-rabbit IgG (1:2000, Abcam), FITC-conjugated anti-mouse IgG (1:1000, Abcam), and Cy3-conjucated anti-mouse IgG (1:1000, Abcam) for 1 hr at room temperature in the dark. Following three washes in PBS for 5 min in dark, slides were mounted using DAPI (4; 6-diamidino-2-phenylindole) mounting solution (Vector Laboratories, Youngstown, OH, USA) according to the manufacturer's instructions. Immunofluorescence was detected by confocal microscopy (LSM 5 Pascal Microscope, Carl Zeiss, Oberkochen, Germany). Control slides were incubated without primary antibodies.

### H&E and Sirius Red staining

After fixation with 4% paraformaldehyde solution in PBS, paraffin embedded liver tissue sections (5 μm in thickness) were stained with H&E for histological analysis and Sirius Red (Sigma-Aldrich) staining according to standard techniques for identifying collagen deposition.

### Hepatocyte differentiation in vitro

After passaging T-MSCs three times, hepatocyte differentiation was induced as follows: 10^6^ T-MSCs were cultured for 7 days on a 100-mm culture dish in DMEM containing 5% FBS, 100 μg/mL streptomycin, 100 U/mL penicillin, 25 ng/mL IGF (R&D Systems Minneapolis, MN, USA), 25 ng/mL HGF (R&D Systems), and 10^−7^ M dexamethasone (Sigma-Aldrich), followed by 14 days in the same medium supplemented with OSM (10 ng/mL, R&D Systems). Culture medium was changed twice a week until day 21. Control cells were cultured without IGF, HGF, dexamethasone, or OSM for 21 days.

### Statistical analysis

Data are expressed as the mean ± standard error of the mean (SEM). One-way or two-way ANOVA was applied for group analysis, and Student's *t*-test was used to identify statistically significant differences in staining (at P < 0.05). All statistical analyses were performed using GraphPad Prism Software (GraphPad Software Inc., San Diego, CA, USA).

## Author Contributions

M.P., H.L. and Y.Y. collected the data. M.P., Y.K. and S.W. analyzed the data. S.W., H.K. and K.R. designed the experiments. H.K. and Y.P. provided human samples. I.J. and K.R. provided the financial support. S.J., J.P. and B.J. provided the administrative support. Y.K., S.W., I.J., H.L. and K.R. prepared the manuscript.
